# Melittin Ameliorates Endotoxin-Induced Acute Kidney Injury by Inhibiting Inflammation, Oxidative Stress, and Cell Death in Mice

**DOI:** 10.1155/2021/8843051

**Published:** 2021-01-04

**Authors:** Jung-Yeon Kim, Jaechan Leem, Hyo-Lim Hong

**Affiliations:** ^1^Department of Immunology, School of Medicine, Catholic University of Daegu, Daegu 42472, Republic of Korea; ^2^Department of Internal Medicine, School of Medicine, Catholic University of Daegu, Daegu 42472, Republic of Korea

## Abstract

Sepsis-related acute kidney injury (AKI) is a worldwide health problem, and its pathogenesis involves multiple pathways. Lipopolysaccharide (LPS) is an endotoxin that induces systemic inflammatory responses. Melittin, a main constituent of bee venom, exerts several biological activities such as antioxidant, anti-inflammatory, and antiapoptotic actions. However, whether melittin protects against endotoxin-induced AKI remains undetermined. Here, we aimed to examine the potential action of melittin on LPS-induced renal injury and explore the mechanisms. We showed that acute renal failure and structural damage after injection of LPS were markedly attenuated by administration of melittin. The peptide also suppressed expression of markers of direct tubular damage in kidneys of the LPS-treated mice. Mechanistically, melittin reduced systemic and renal levels of cytokines and inhibited renal accumulation of immune cells with concomitant suppression of nuclear factor kappa-B pathway. Increased amounts of lipid peroxidation products after LPS treatment were largely decreased by melittin. Additionally, the peptide decreased expression of nicotinamide adenine dinucleotide phosphate oxidase 4 and enhanced nuclear factor erythroid-2-related factor 2-mediated antioxidant defenses. Moreover, melittin inhibited apoptotic and necroptotic cell death after LPS treatment. Lastly, we showed that melittin improved the survival rate of LPS-injected mice. These results suggest that melittin ameliorates endotoxin-induced AKI and mortality through inhibiting inflammation, oxidative injury, and apoptotic and necroptotic death of tubular epithelial cells.

## 1. Introduction

Sepsis is a serious medical condition triggered by abnormal immune responses to infection and remains a main global cause of death in hospitals [1]. Acute kidney injury (AKI) is frequently occurred comorbidity of sepsis and is correlated with increased mortality in critically ill patients [2]. Standard therapy for patients suffering from sepsis-related renal injury includes rapid administration of antibiotics and avoidance of hypotension through adequate fluid resuscitation or use of vasopressors [3]. However, the current therapy has limitations, and the development of novel pharmacological agents which are aimed at safely and effectively controlling sepsis-related AKI will provide a more promising therapeutic approach for preventing the serious medical condition. The mechanism of sepsis-related AKI is multifactorial and still remains unclear. Pathogen-associated molecular patterns (PAMPs) are produced by microorganisms and are detected by pattern recognition receptors. During sepsis, they are released into blood circulation [3]. Among PAMPs, lipopolysaccharide (LPS) is an endotoxin produced by gram-negative bacteria. This inflammatory mediator stimulates many different types of cells including renal tubular epithelial cells and immune cells through interacting with Toll-like receptors (TLRs), resulting in the induction of oxidative stress, inflammation, and cell death [3–5].

Over the centuries, bee venom therapy has been applied to the management of acute and chronic human diseases [6]. The toxin contains a variety of bioactive peptides and enzymes. Among them, melittin is a polypeptide constituting about 50% of the dry weight of the toxin [7]. Emerging evidence suggests that the peptide has multiple biological functions including antioxidant, anti-inflammatory, and antibacterial actions [8, 9]. A recent study has demonstrated that melittin ameliorated renal injury and fibrosis after unilateral ureteral obstruction in mice [10]. However, whether melittin protects against LPS-induced AKI remains undetermined. In this study, we evaluated the action of melittin on endotoxin-induced renal injury and explored the mechanisms.

## 2. Materials and Methods

### 2.1. Materials

Melittin was purchased from Enzo Life Sciences (Farmingdale, NY, USA), and LPS was acquired from Sigma-Aldrich (St. Louis, MO, USA). Creatinine and blood urea nitrogen (BUN) assay kits were acquired from Bioassay Systems (Hayward, CA, USA) and Asan Pharmaceutical (Seoul, Korea), respectively. Enzyme-linked immunosorbent assay (ELISA) kits for tumor necrosis factor-*α* (TNF-*α*) and interleukin-6 (IL-6) were obtained from R&D Systems (Minneapolis, MN, USA). The lipid peroxidation colorimetric/fluorometric assay kit was obtained from Sigma-Aldrich. Lotus tetragonolobus lectin (LTL) conjugated with fluorescein was acquired from Vector Laboratories (Burlingame, CA, USA). Primary antibodies against inhibitor *κ*B-*α* (I*κ*B-*α*), p-I*κ*B-*α*, nuclear factor-*κ*B (NF-*κ*B) p65, p-NF-*κ*B p65, cleaved poly(ADP-ribose) polymerase-1 (PARP-1), cleaved caspase-3, p53, receptor-interacting serine/threonine protein kinase 1 (RIPK1), RIPK3, mixed-lineage kinase domain-like protein (MLKL), p-MLKL, or glyceraldehyde-3-phosphate dehydrogenase (GAPDH) were obtained from Cell Signaling (Danvers, MA, USA). Primary antibodies against Mac-2, CD4, 4-hydroxynonenal (4-HNE), TNF-*α*, IL-6, kidney injury molecule-1 (Kim-1), and nuclear factor erythroid-2-related factor 2 (Nrf2) were obtained from Abcam (Cambridge, MA, USA). Antineutrophil gelatinase-associated lipocalin (NGAL) and anti-Bax primary antibodies were acquired from Santa Cruz Biotechnology (Santa Cruz, CA, USA). The primary antibody against nicotinamide adenine dinucleotide phosphate oxidase 4 (NOX4) was obtained from Novus Biologicals (Littleton, CO, USA), and the primary antibody against heme oxygenase 1 (HO-1) was acquired from Enzo Life Sciences. Anti-lamin B1 primary antibody was purchased from Invitrogen (Carlsbad, CA, USA). The nuclear protein extraction kit was obtained from Thermo Fisher Scientific (Waltham, MA, USA), and the Bradford protein assay kit was acquired from Bio-Rad Laboratories (Hercules, CA, USA). The RNeasy Mini Kit was acquired from Qiagen (Valencia, CA, USA), and the High-Capacity cDNA Reverse Transcription Kit was obtained from Applied Biosystems (Foster City, CA, USA). The Power SYBR Green PCR Master Mix was acquired from Applied Biosystems. The in situ cell death detection kit was obtained from Roche Diagnostics (Indianapolis, IN, USA), and the Lightshift® Chemiluminescent EMSA Kit was obtained from Thermo Fisher Scientific.

### 2.2. Animals

Animal experiments were carried out on male C57BL/6 N mice (8 weeks of age; Samtako Bio Korea, Osan, Korea). Mice were housed at 22 ± 2°C in 12-hour day/night cycles and had free access to standard rodent diet and water. The Institutional Animal Care and Use Committee of the Daegu Catholic University Medical Center approved all experimental procedures.

### 2.3. Protocol of LPS-Induced AKI

Mice were arbitrarily categorized into three groups (*n* = 8 per each group): vehicle-injected group (Veh), LPS-injected group (LPS), and group treated with LPS plus melittin (LPS+Mel). Mice in the LPS group were intraperitoneally injected with LPS (10 mg/kg). Mice in the Veh group were subjected to an intraperitoneal injection of an equal volume of sterile saline. Melittin (0.01 mg/kg) was intraperitoneally administered to mice in the LPS+Mel group 1 hour before injection of LPS. The doses of melittin and LPS used in this study were selected based on the results of previous studies [10–12]. At 24 hours after injection of LPS, all mice were sacrificed. Kidneys were promptly isolated, and blood sample collection was carried out by cardiac puncture. For survival experiments, mice were arbitrarily categorized into three groups (*n* = 10 per each group; Veh, LPS, and LPS+Mel). Mice were intraperitoneally injected with melittin (0.01 mg/kg) 1 hour before injection of LPS (20 mg/kg). Survival was monitored until 96 hours after LPS treatment.

### 2.4. Biochemical Analyses

Separation of whole blood into plasma was carried out using a centrifugation method. Renal function was assessed by evaluating plasma concentrations of creatinine and BUN, which were performed using creatinine and BUN assay kits, respectively. Plasma concentrations of TNF-*α* and IL-6 were analyzed using ELISA kits. Amounts of malondialdehyde (MDA), a lipid peroxidation product, in kidneys were analyzed using a lipid peroxidation colorimetric/fluorometric assay kit.

### 2.5. Histological Analyses, Immunofluorescence, and Immunohistochemisty (IHC)

Kidneys were fixed in 4% paraformaldehyde. The tissues were then dehydrated, cleared, embedded in paraffin blocks, and sectioned. After sectioning, hematoxylin and eosin (H&E) and periodic acid-Schiff (PAS) stains were performed on the thin sections. Tubular injury was assessed in 10 arbitrarily selected nonoverlapping fields (400x magnification) of PAS-stained sections for each kidney. The degree of injury was scored based on the percentage of damaged area: score 0, no tubular injury; score 1, ≤10% of tubules injured; score 2, 11–25% of tubules injured; score 3, 26–45% of tubules injured; score 4, 46–75% of tubules injured; score 5, 76–100% of tubules injured [13, 14]. Brush border of proximal tubules was identified by staining with LTL conjugated with fluorescein. For IHC, the kidney sections were probed with primary antibodies against NGAL, Kim-1, Mac-2, CD4, or 4-HNE. After washing, the sections were incubated with a secondary antibody. Nuclear counterstain was performed using hematoxylin. The percentage of positive staining for specific targets was analyzed in 10 arbitrarily selected fields (400x magnification) for each kidney using the i-Solution DT software (IMTechnology, Vancouver, BC, Canada). The number of cells stained with Mac-2 or CD4 was computed in 10 arbitrarily selected fields (400x magnification) for each kidney.

### 2.6. Western Blotting

Proteins were extracted from tissues with a lysis buffer. Nuclear fraction from tissues was isolated using a nuclear protein extraction kit. The protein samples were separated using sodium dodecyl sulfate-polyacrylamide gel electrophoresis. The separated proteins were transferred onto a membrane, which was then probed with primary antibodies against NGAL, TNF-*α*, IL-6, I*κ*B-*α*, p-I*κ*B-*α*, NF-*κ*B p65, p-NF-*κ*B p65, NOX4, Nrf2, HO-1, cleaved caspase-3, cleaved PARP-1, p53, Bax, RIPK1, RIPK3, MLKL, p-MLKL, lamin B1, or GAPDH. After washing, the membrane was probed with a secondary antibody. Lamin B1 and GAPDH were used as a loading control. The ChemiDoc^TM^ XRS+ System (Bio-Rad Laboratories) was used for detecting chemiluminescent signals.

### 2.7. Real-Time Reverse Transcription-Polymerase Chain Reaction (RT-PCR)

Total RNA was extracted from tissues using the RNeasy Mini Kit. The High-Capacity cDNA Reverse Transcription Kit was used to synthesize cDNA. Real-time RT-PCR was conducted using the Power SYBR Green PCR Master Mix and the Applied Biosystems 7500 Real-Time PCR System.  Table 1 shows primer sequences used in this study. Relative expression of specific gene was calculated by the 2^-*ΔΔ*CT^ method, using GAPDH as a reference gene.

### 2.8. TdT-Mediated dUTP Nick End Labeling (TUNEL) Staining

Tubular cell apoptosis in the kidney sections was identified by TUNEL staining using the in situ cell death detection kit according to the manufacturer's instructions. In brief, the sections were deparaffinized, rehydrated, and permeabilized. Then, the sections were probed with a TUNEL reaction mixture. The 4′,6-diamidino-2-phenylindole (DAPI) was applied to visualize nuclei. Cells stained with TUNEL were counted in 10 arbitrarily selected nonoverlapping (400x magnification) for each kidney.

### 2.9. Electrophoretic Mobility Shift Assay (EMSA)

EMSA was performed using the Lightshift® Chemiluminescent EMSA Kit according to the manufacturer's instructions. The chemiluminescence of biotin-labelled DNA was detected using the Chemidoc XRS+ System (Bio-Rad Laboratories). The NF-*κ*B oligonucleotide probe (5′-AGT TGA GGG GAC TTT CCC AGG C-3′) was end labelled with digoxigenin ddUTP.

### 2.10. Statistical Analysis

The results are presented as the mean ± standard error of the mean (SEM). Significant differences among groups were determined using one-way analysis of variance (ANOVA) followed by Bonferroni's post hoc tests. Survival analysis was performed using the Kaplan-Meier method. A *p* value less than 0.05 was considered to be statistically significant.

## 3. Results

### 3.1. Melittin Dampened LPS-Induced Renal Dysfunction and Structural Damage

**Figure 1:**
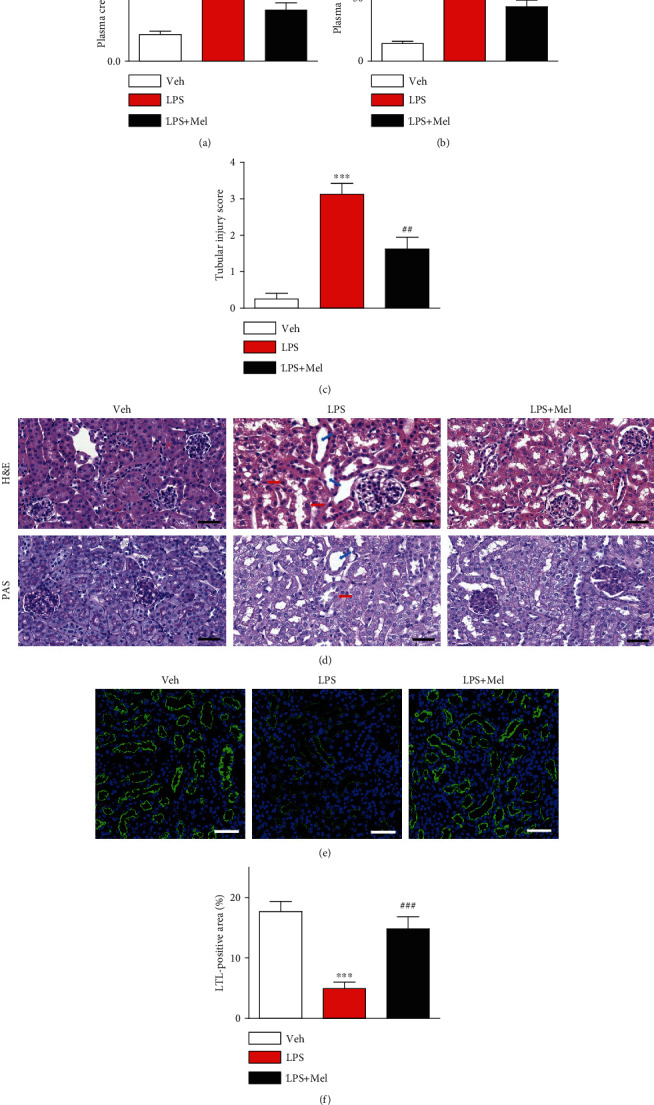
Effects of melittin on renal function and structure in LPS-injected mice. Melittin (Mel; 0.01 mg/kg) was administered intraperitoneally to mice 1 hour prior to LPS treatment (10 mg/kg). Mice in control group were subjected to an intraperitoneal injection of vehicle (saline). (a) Plasma creatinine levels. (b) Plasma BUN levels. (c) Tubular injury score. (d) H&E and PAS staining of kidney tissues from the indicated group. Blue arrows indicate tubular dilatation. Red arrows indicate swelling of renal tubular epithelial cells. Scale bars: 50 *μ*m. (e) Immunofluorescent staining with fluorescein-conjugated LTL of kidney tissues. Scale bars: 50 *μ*m. (f) Average percentage of positive staining for LTL per field. *n* = 8 per each group. ^∗∗∗^*p* < 0.001 compared with the vehicle-injected group (Veh). ^##^*p* < 0.01 or ^###^*p* < 0.001 compared with the LPS-injected group (LPS).

LPS-treated mice exhibited a profound decline of renal function, as represented by increased plasma concentrations of creatinine and BUN, at 24 hours after injection of LPS (Figures 1(a) and 1(b)). H&E and PAS staining showed that LPS-injected mice displayed histopathological alterations, including tubular dilatation and swelling of tubular epithelial cells (Figures 1(c) and 1(d)). Markedly reduced LTL staining after LPS treatment was also observed, indicating that exposure of LPS induced brush border loss in proximal tubules (Figures 1(e) and 1(f)). However, the renal dysfunction and structural damage induced by the endotoxin were significantly ameliorated by administration of melittin.

**Figure 2:**
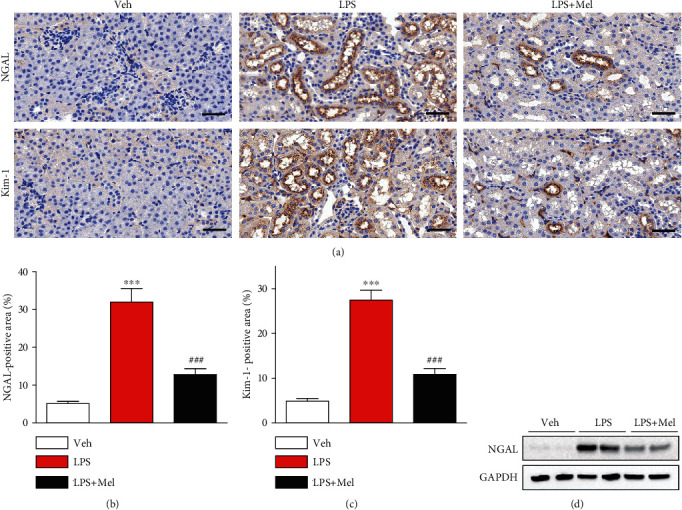
Effects of melittin on expression of tubular injury markers after LPS treatment. (a) Immunohistochemical staining of kidney tissues from the indicated group with primary antibodies against NGAL or Kim-1. Scale bars: 50 *μ*m. (b) Average percentage of positive staining for NGAL per field. (c) Average percentage of positive staining for Kim-1 per field. (d) Western blotting of NGAL in kidneys. ^∗∗∗^*p* < 0.001 compared with the vehicle-injected group (Veh). ^###^*p* < 0.001 compared with the LPS-injected group (LPS).

Next, immunohistochemical staining of kidney sections with anti-NGAL or anti-Kim-1 antibody was carried out to further explore the action of melittin on tubular injury in LPS-injected mice. We found that melittin significantly reduced expression of the tubular injury markers after LPS treatment (Figures 2(a)–2(c)). Western blotting also revealed that increased NGAL protein levels in LPS-injected mice were largely suppressed by melittin (Figure 2(d)).

### 3.2. Melittin Suppressed LPS-Induced Inflammation

**Figure 3:**
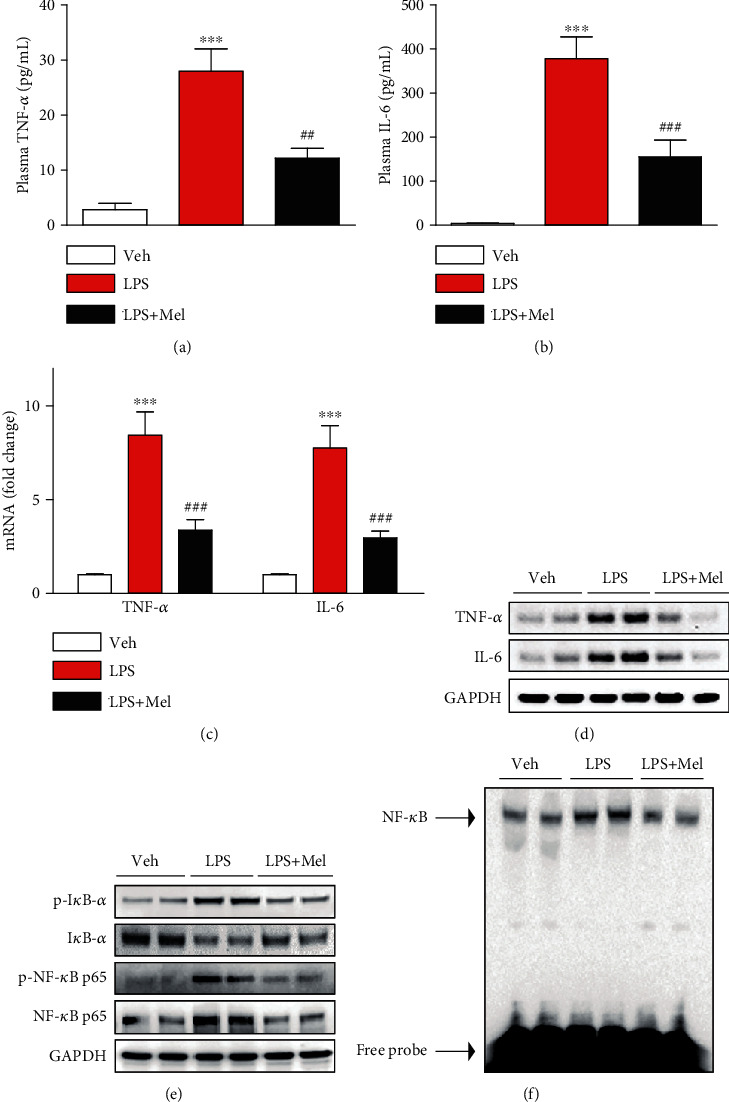
Effects of melittin on plasma and renal levels of cytokines and NF-*κ*B signaling cascade after LPS treatment. (a) Plasma TNF-*α* levels. (b) Plasma IL-6 levels. (c) The mRNA levels of TNF-*α* and IL-6 in kidney tissues. (d) Western blotting of TNF-*α* and IL-6 in kidney tissues. (e) Western blotting of I*κ*B-*α*, p-I*κ*B-*α*, NF-*κ*B p65, and p-NF-*κ*B p65 in kidney tissues. (f) Electrophoretic mobility shift assay for NF-*κ*B DNA-binding activity in kidney tissues. ^∗∗∗^*p* < 0.001 compared with the vehicle-injected mice (Veh). ^##^*p* < 0.01 or ^###^*p* < 0.001 compared with the LPS-injected group (LPS).

**Figure 4:**
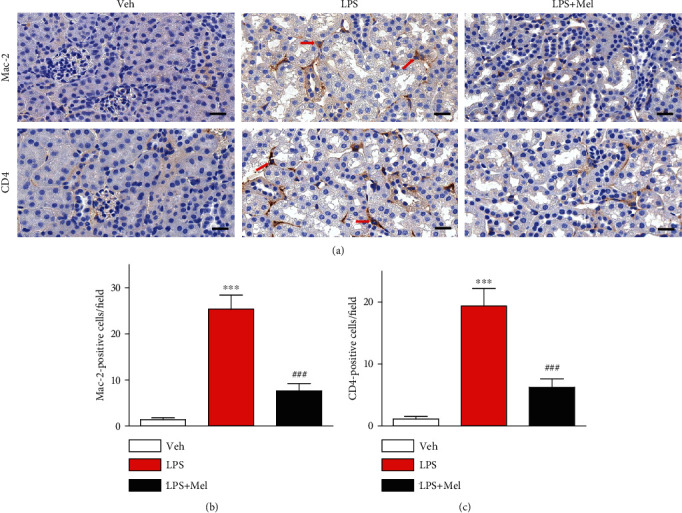
Effects of melittin on immune cell accumulation after LPS treatment. (a) Immunohistochemical staining of kidney tissues from the indicated group with anti-Mac-2 or anti-CD4 antibody. Red arrows indicate cells stained with Mac-2 or CD4. Scale bars: 20 *μ*m. (b) Average number of cells stained with Mac-2 per field. (c) Average number of cells stained with CD4 per field. ^∗∗∗^*p* < 0.001 compared with the vehicle-injected group (Veh). ^###^*p* < 0.001 compared with the LPS-injected group (LPS).

Endotoxin can trigger inflammatory activation by increasing the secretion of numerous inflammatory mediators from immune cells [3]. It has been shown that melittin exerts anti-inflammatory activities [8]. To investigate the action of melittin on endotoxin-induced inflammatory responses, we measured plasma concentrations of TNF-*α* and IL-6 in all groups. Administration of melittin significantly reduced plasma concentrations of both cytokines after LPS treatment (Figures 3(a) and 3(b)). Increased mRNA and protein levels of these cytokines in kidneys were also largely reduced by melittin (Figures 3(c) and 3(d)). Next, we evaluated the effects of melittin on endotoxin-induced NF-*κ*B activation. Melittin markedly decreased p-I*κ*B-*α* expression and increased I*κ*B-*α* expression in kidneys of LPS-injected mice, indicating that a decrease in I*κ*B-*α* phosphorylation led to increased stabilization of I*κ*B-*α* after melittin treatment (Figure 3(e)). Levels of NF-*κ*B p65 and its phosphorylated form in kidneys were also markedly decreased by melittin (Figure 3(e)). To further examine the action of the peptide on DNA-binding activity of NF-*κ*B, nuclear extracts were analyzed by EMSA. Increased DNA-binding activity of NF-*κ*B was observed in kidneys of LPS-injected mice (Figure 3(f)). However, this increased binding activity was largely suppressed by melittin. Immunohistochemical staining also showed that melittin largely reduced number of cells stained with Mac-2 or CD4 in kidneys of LPS-treated mice, indicating that the peptide suppresses infiltration of immune cells into the injured kidneys (Figures 4(a)–4(c)).

### 3.3. Melittin Inhibited LPS-Induced Oxidative Damage

**Figure 5:**
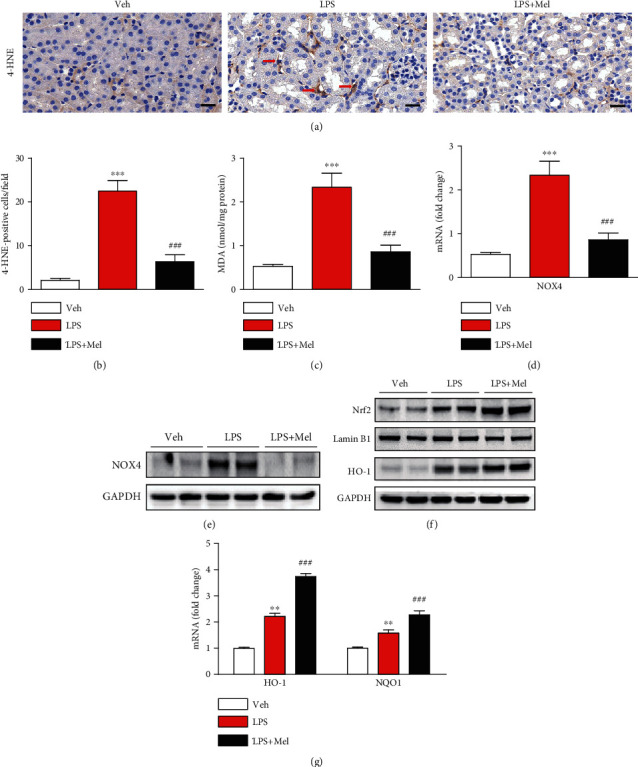
Effects of melittin on oxidative damage and expression of prooxidant and antioxidant proteins after LPS treatment. (a) Immunohistochemical staining of kidney tissues from the indicated group with anti-4-HNE antibody. Red arrows indicate cells stained with 4-HNE. Scale bars: 20 *μ*m. (b) Average number of cells stained with 4-HNE per field. (c) Renal MDA levels. (d) The mRNA level of NOX4 in kidney tissues. (e) Western blotting of NOX4 in kidney tissues. (f) Western blotting of Nrf2 and HO-1 in kidney tissues. (g) The mRNA levels of HO-1 and NQO1 in kidney tissues. ^∗∗^*p* < 0.01 or ^∗∗∗^*p* < 0.001 compared with the vehicle-injected group (Veh). ^####^*p* < 0.001 compared with LPS-injected group (LPS).

Besides inflammation, oxidative stress also plays a critical role in the pathophysiology of endotoxin-induced renal injury [4]. To next evaluate the effects of melittin on oxidative stress, we stained the kidney sections with a 4-HNE antibody to detect lipid peroxidation products. We found that the number of 4-HNE-positive cells was largely increased in kidneys of LPS-injected mice compared to control mice, which was significantly reduced by melittin (Figures 5(a) and 5(b)). Renal MDA levels were also significantly reduced by melittin after LPS treatment (Figure 5(c)).

NOX4 is one of main sources of reactive oxygen species (ROS) in LPS-induced AKI [15, 16]. We found that mRNA and protein levels of NOX4 were largely decreased by melittin in kidneys of LPS-injected mice (Figures 5(d) and 5(e)). Additionally, treatment with LPS also led to nuclear translocation of Nrf2 and increased protein level of HO-1, its target gene, in kidneys (Figure 5(f)). Interestingly, administration of melittin further enhanced the nuclear translocation of Nrf2 and protein expression of HO-1. Renal mRNA level of NAD(P)H:quinone oxidoreductase 1 (NQO1), another target gene of Nrf2, as well as HO-1 was also significantly enhanced by melittin, indicating that the peptide further activated Nrf2-dependent antioxidant response pathway (Figure 5(g)).

### 3.4. Melittin Attenuated LPS-Induced Apoptosis

**Figure 6:**
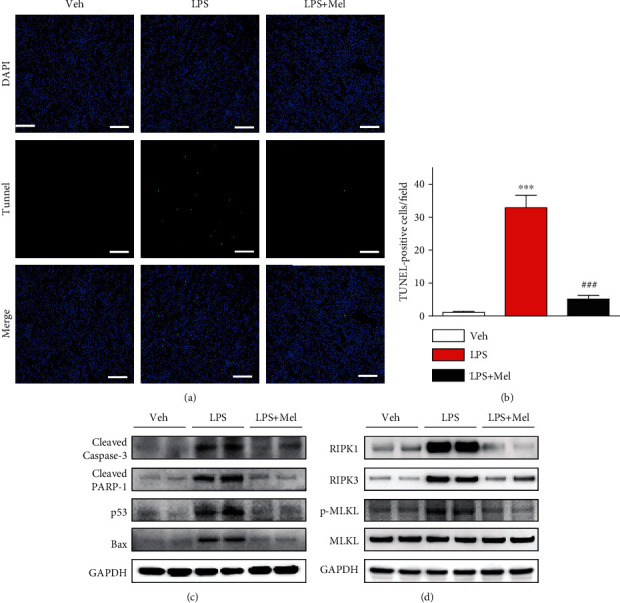
Effects of melittin on tubular cell apoptosis and necroptosis after LPS treatment. (a) TUNEL staining of kidney tissues from the indicated group. Scale bars: 100 *μ*m. (b) Average number of cells stained with TUNEL per field. (c) Western blotting of cleaved caspase-3, cleaved PARP-1, p53, and Bax in kidney tissues. (d) Western blotting of RIPK1, RIPK3, p-MLKL, and MLKL in kidney tissues. ^∗∗∗^*p* < 0.001 compared with the vehicle-injected group (Veh). ^###^*p* < 0.001 compared with the LPS-injected group (LPS).

Apoptosis also contributes to the pathophysiology of endotoxin-induced renal injury [5]. Thus, we investigated the action of melittin on endotoxin-induced apoptosis. LPS-injected mice exhibited a profound increase in number of cells stained with TUNEL in kidneys (Figures 6(a) and 6(b)). However, melittin significantly reduced the number of TUNEL-stained cells. Additionally, increased cleavage of caspase-3 and PARP-1, its substrate, after LPS treatment was markedly attenuated by melittin (Figure 6(c)). We also found that melittin reduced protein levels of p53 and Bax (Figure 6(c)).

Besides apoptosis, recent studies have shown that necroptosis, another form of cell death, also plays a critical role in AKI [17]. We observed that treatment with LPS markedly increased protein expression of RIPK1, RIPK3, and p-MLKL, indicating that LPS induces necroptotic cell death (Figure 6(d)). However, these changes were significantly reversed by melittin.

### 3.5. Melittin Improved Survival Rate in LPS-Injected Mice

**Figure 7:**
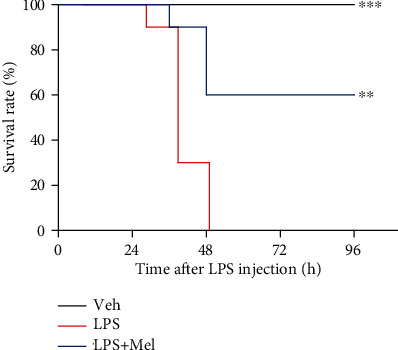
Effects of melittin on survival rate after LPS treatment. Melittin (0.01 mg/kg) intraperitoneally administered to mice 1 hour prior to injection of LPS (20 mg/kg). Mice in control group were subjected to an intraperitoneal injection of vehicle (saline). *n* = 10 per each group. ^∗∗^*p* < 0.01 or ^∗∗∗^*p* < 0.001 compared with the LPS-injected group (LPS).

Lastly, to evaluate the effects of melittin on survival rate of LPS-injected mice, melittin at a dose of 0.01 mg/kg intraperitoneally administered to mice 1 hour before injection of LPS at a dose of 20 mg/kg. We found that melittin treatment significantly enhanced the survival rate of LPS-injected mice (Figure 7).

## 4. Discussion

**Figure 8:**
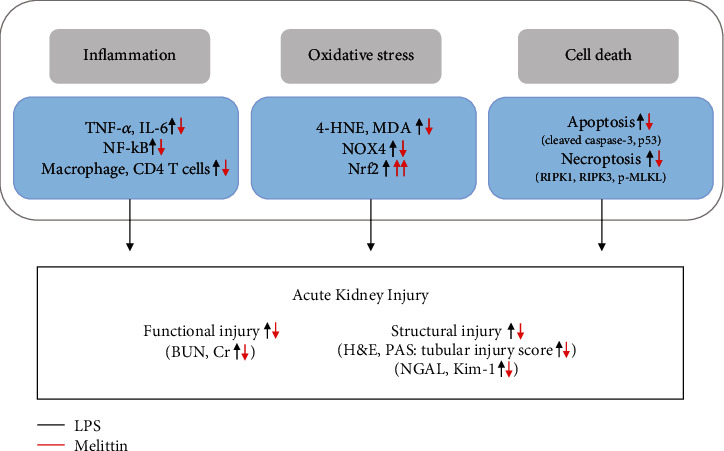
Schematic summary of the results of the present study. LPS-induced renal injury was dampened by melittin. These protective effects of the peptide were attributed to inhibition of inflammation, oxidative damage, and apoptotic and necroptotic death of tubular epithelial cells.

In this study, we evaluated the potential action of melittin on LPS-induced renal injury and investigated its mechanisms. Our data showed that administration of melittin ameliorated AKI and improved survival rate after LPS treatment. The favorable action of the peptide was attributed to inhibition of inflammation, oxidative damage, and tubular cell death (Figure 8). Our data reveal a novel beneficial effect of melittin against LPS-induced renal injury.

Sepsis-related AKI is closely correlated with poor clinical outcome in critically ill patients [2]. However, there are currently no pharmacological treatments for sepsis-related renal injury. Recently, we showed the beneficial action of bee venom and its component apamin on LPS-induced acute renal failure and structural damage [12, 18]. However, the effects of melittin, the major component of bee venom, against LPS-induced AKI have not yet been reported. In this study, we demonstrated that melittin dampened an acute decline in renal function, as represented by reduced plasma creatinine and BUN levels, and structural damage such as tubular dilatation, swelling of tubular epithelial cells, and brush border loss. Additionally, increased expression of Kim-1 and NGAL, tubular injury markers, was also largely attenuated by the peptide. Taken together, these findings suggest that melittin displays renoprotective effects against endotoxin-induced functional and structural injury.

PAMPs play a key role in the regulation of host immune response to infection and are detected by pattern-recognition receptors [19]. LPS is a well-known PAMP and is produced by gram-negative bacteria. During bacterial sepsis, this inflammatory mediator interacts with TLRs that are expressed on the surface of many different types of cells including renal tubular epithelial cells and immune cells, leading to the abnormal secretion of cytokines [3]. In this study, we analyzed plasma and renal concentrations of cytokines to evaluate whether melittin exerts anti-inflammatory effects. Plasma and local tissue concentrations of TNF-*α* and IL-6 were largely increased after injection of LPS. However, melittin suppressed the levels of cytokines, suggesting that the peptide suppressed systemic and local inflammatory responses induced by LPS. Given that NF-*κ*B is a critical transcriptional modulator that regulates production of cytokines in the damaged kidneys [20], we next evaluated its effects on NF-*κ*B signaling cascade. Melittin significantly decreased phosphorylated levels of I*κ*B-*α* and NF-*κ*B p65 after LPS treatment. Moreover, DNA-binding activity of NF-*κ*B p65 was suppressed by melittin. In line with our results, previous studies suggest that the peptide displays anti-inflammatory activities against a variety of inflammatory diseases [8]. It was reported that the administration of melittin suppressed the production of inflammatory cytokines in unilateral ureteral obstruction-induced renal injury [10]. The peptide attenuated endotoxin-induced NF-*κ*B activation in human keratinocytes and monocytes [21, 22]. Melittin also suppressed the production of inflammatory cytokines in atherosclerotic mice induced by LPS and high-fat diet [22]. In addition, the peptide ameliorated D-galactosamine and endotoxin-induced acute hepatic failure through suppressing NF-*κ*B signaling [23]. It has been known that massive infiltration of immune cells into the injured kidneys is frequently observed after LPS treatment [12]. Infiltrating immune cells can produce various cytokines and aggravate inflammatory responses in kidneys. In this study, melittin alleviated endotoxin-induced accumulation of immune cells in kidney tissues. It was recently reported that the peptide inhibited accumulation of mast cells and T lymphocytes in the skin lesions of experimental atopic dermatitis [11, 24].

In addition to inflammation, oxidative stress also plays a critical role in sepsis-related renal injury [4, 25]. Previous studies have reported that sepsis-related AKI is closely correlated with ROS overproduction [26, 27]. In the present study, LPS-injected mice exhibited increased renal amounts of lipid peroxidation products, which were significantly suppressed by melittin. We also observed that melittin markedly reduced renal NOX4 expression. Because NOX4 is one of main ROS generators in the pathophysiology of endotoxin-induced renal injury [15, 16], decreased expression of the prooxidant enzyme by melittin may decrease ROS production in kidneys. Nrf2 is a crucial transcription factor that modulates expression of antioxidant genes and provides a critical cellular defense against oxidative stress [28, 29]. Increased ROS can trigger Nrf2 response [30]. In this study, we found that melittin increased nuclear translocation of Nrf2 with concurrent upregulation of its target genes, HO-1 and NQO1, in the kidneys of LPS-injected mice. A previous study also reported that melittin ameliorated myocarditis by activating Nrf2 in mice [31]. Collectively, these findings suggest that the regulation of prooxidant and antioxidant systems by melittin was involved in its beneficial action against LPS-induced AKI.

It has been known that Nrf2 pathway suppresses NF-*κ*B activation and vice versa [32]. However, in our study, we observed that NF-*κ*B and Nrf2 signaling pathways were activated by LPS treatment at the same time. In good agreement with our observations, previous studies also reported the simultaneous activation of both signaling pathways in LPS-injected mice [33–35]. LPS-induced Nrf2 activation may be a protective mechanism against NF-*κ*B-mediated inflammatory responses. However, the degree of Nrf2 activation does not appear to sufficiently inhibit NF-*κ*B signaling. Interestingly, melittin treatment further enhanced the LPS-induced Nrf2 activation, leading to the suppression of NF-*κ*B signaling pathway. Consistent with these findings, mRNA and protein expression of HO-1 were also significantly enhanced by melittin. However, as HO-1 expression can be regulated by other transcription factors [36, 37], we cannot exclude the possibility that Nrf2-independent pathways are also involved in the modulation of HO-1.

Apoptosis also contributes to the pathophysiology of sepsis-related renal injury [5]. Exposure of renal tubular epithelial cells to LPS results in profound apoptosis [38]. Administration of a pan-caspase inhibitor attenuated apoptosis and renal injury in endotoxin-induced renal injury [39]. In this study, LPS-treated mice displayed a profound increase in number of cells stained with TUNEL with concomitant activation of caspase-3 in kidneys. However, the detrimental effects of LPS were markedly alleviated by melittin. Mechanistically, melittin reduced renal levels of p53 and Bax, its transcriptional target gene, after LPS treatment, indicating that the peptide suppressed p53-dependent apoptotic pathway. Because tubular cell apoptosis is an important contributing factor for sepsis-related renal injury [5], our findings suggest that the beneficial action of melittin against endotoxin-induced renal damage is, at least partially, attributed to its antiapoptotic activity. Previous researches have focused on melittin's antitumor activity [40]. However, recent studies reported that the peptide exerts strong antiapoptotic effects in various types of normal cells [41, 42] and tissues [23, 31].

Necroptosis is a programmed form of necrosis and is an important pathogenic process in AKI [17]. Emerging evidence suggests that necroptosis is critically involved in renal ischemia-reperfusion injury [43], cisplatin-induced nephrotoxicity [44], and sepsis-related renal injury [45]. During necroptosis, RIPK1 interacts with RIPK3 to form a multiprotein complex, which regulates the phosphorylation of MLKL. Phosphorylated MLKL induces plasma membrane rupture and thereby leads to releasing of the intracellular components, resulting in the induction of inflammatory responses. In this study, expression of RIPK1, RIPK3, and phosphorylated MLKL was increased after LPS treatment, which was markedly attenuated by melittin. Collectively, these findings indicate that melittin inhibited LPS-induced necroptotic cell death.

## 5. Conclusions

These results suggest that melittin protects against endotoxin-induced renal injury and mortality via suppression of inflammatory responses, oxidative stress, and apoptotic and necroptotic cell death. The peptide has been also shown to have a potent antibacterial property [9]. Therefore, melittin may be a potential therapeutic option for preventing the renal complication of sepsis.

## Figures and Tables

**Table 1:** Gene-specific primer sets used in the present study.

Target genes	Primer sequences	Accession no.
TNF-*α*	F: 5′-GACGTGGAACTGGCAGAAGAG-3′	NM_013693
	R: 5′-CCGCCTGGAGTTCTGGAA-3′	
IL-6	F: 5′-CCAGAGATACAAAGAAATGATGG-3′	NM_031168
	R: 5′-ACTCCAGAAGACCAGAGGAAAT-3′	
NOX4	F: 5′-GAACCCAAGTTCCAAGCTCATT-3′	NM_015760
	R: 5′-GGCACAAAGGTCCAGAAATCC-3′	
HO-1	F: 5′-TCAAGGCCTCAGACAAATCC-3′	NM_010442
	R: 5′-ACAACCAGTGAGTGGAGCCT-3′	
NQO1	F: 5′-AATGGGCCAGTACAATCAGG-3′	NM_008706
	R: 5′-CCAGCCCTAAGGATCTCTCC-3′	
GAPDH	F: 5′-ACTCCACTCACGGCAAATTC-3′	NM_001289726
	R: 5′-TCTCCATGGTGGTGAAGACA-3′	

## Data Availability

The data used to support the findings of this study are available from the corresponding authors upon request.
